# JACKS: joint analysis of CRISPR/Cas9 knockout screens

**DOI:** 10.1101/gr.238923.118

**Published:** 2019-03

**Authors:** Felicity Allen, Fiona Behan, Anton Khodak, Francesco Iorio, Kosuke Yusa, Mathew Garnett, Leopold Parts

**Affiliations:** 1 Wellcome Sanger Institute, Wellcome Genome Campus, Hinxton, Cambridgeshire, CB10 1SA, United Kingdom;; 2 Department of Computer Science, University of Tartu, Tartu 50409, Estonia

## Abstract

Genome-wide CRISPR/Cas9 knockout screens are revolutionizing mammalian functional genomics. However, their range of applications remains limited by signal variability from different guide RNAs that target the same gene, which confounds gene effect estimation and dictates large experiment sizes. To address this problem, we report JACKS, a Bayesian method that jointly analyzes screens performed with the same guide RNA library. Modeling the variable guide efficacies greatly improves hit identification over processing a single screen at a time and outperforms existing methods. This more efficient analysis gives additional hits and allows designing libraries with a 2.5-fold reduction in required cell numbers without sacrificing performance compared to current analysis standards.

CRISPR/Cas9 knockout screens can assess the influence of every gene's knockout on any selectable cellular trait in a single assay (Shalem et al. 2014; Wang et al. 2014). The guide RNA (gRNA) libraries used in these experiments typically contain several gRNAs per gene, each steering the Cas9 protein to inflict a loss-of-function mutation. Genes required for the selected trait are mapped by introducing the gRNA library into cells, applying selection, sequencing the gRNA locus, and processing the data using methods such as MAGeCK (Li et al. 2014) or BAGEL (Hart and Moffat 2016).

A central source of confounding in the analysis of screen outputs is conflicting evidence from alternative gRNAs targeting the same gene, caused by different gRNA efficacies (Chuai et al. 2016). This variability has been linked to a range of technical and biological factors (Bae et al. 2014; Doench et al. 2014; Sanjana et al. 2014; Moreno-Mateos et al. 2015; Farasat and Salis 2016; Horlbeck et al. 2016), and although several gRNA efficacy estimation algorithms have been proposed (Doench et al. 2014, 2016; Wang et al. 2014; Chari et al. 2017; Rahman and Rahman 2017), their predictive ability remains limited (Haeussler et al. 2016; Labuhn et al. 2017). As a result, screens still use five or more gRNAs per gene, and at least three replicates are recommended (Ong et al. 2017), rendering the required scale a bottleneck for systematic assessment of gene function, particularly in short-term primary cultures and for assessing genetic interactions.

To overcome this issue, we present joint analysis of CRISPR/Cas9 knockout screens (JACKS), a Bayesian method that models gRNA efficacies in multiple screens that use the same gRNA library. We show that JACKS estimates reproducible gRNA efficacies, which leads to improvements in gene essentiality quantification and advances over existing methods. The more efficient inference allows scaling down library sizes while maintaining competitive performance.

## Results

**Figure 1.**
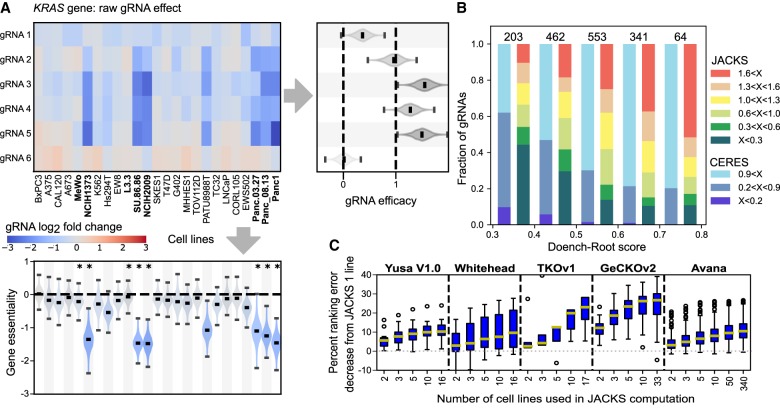
Joint analysis of several CRISPR/Cas9 knockout screens. (*A*) JACKS inferred decomposition of median-normalized log_2_ fold change (heatmap) for six gRNAs targeting the *KRAS* gene (*y*-axis, GeCKOv2 library) in 25 cancer cell lines from Aguirre et al. (2016) (*x*-axis). The inferred gRNA efficacies and gene essentialities (with uncertainty) are displayed to the *right* and *below* the heatmap, respectively. Lines with *KRAS* driver mutations are highlighted in bold and indicated with an asterisk. (*B*) Fraction of gRNAs (*y*-axis) targeting Hart essential genes (Hart et al. 2014) in each range of Doench–Root score (Doench et al. 2016) (*x*-axis) for specified ranges of CERES and JACKS inferred gRNA efficacy scores (“X”; colors). Number of gRNAs in each column is marked *above* the bar. (*C*) Percentage of ranking error (fraction of area above the ROC curve below 0.2 false-positive rate; Methods) decrease (*y*-axis; median, quartiles, and 95th deciles marked in box plot) for increasing number of experiments in JACKS model (*x*-axis) for five different libraries.

JACKS models log_2_ fold changes of gRNA read counts between treatment and control conditions as a product of treatment-dependent gene essentiality and treatment-independent gRNA efficacy (Fig. 1A). We obtain approximate posterior probability distributions for these components, while accounting for experimental noise (Methods), and can use a negative control set to build a null distribution of gene essentialities for *P*-value derivation. In the evaluations below, we applied JACKS to data from pooled knockout screens performed with the Yusa v1.0 (Tzelepis et al. 2016; Iorio et al. 2018), Whitehead (Wang et al. 2015, 2017), Toronto Knockout v1.0 (TKOv1) (Hart et al. 2015), GeCKOv2 (Sanjana et al. 2014; Aguirre et al. 2016), and Avana (Meyers et al. 2017) gRNA libraries.

### JACKS infers reproducible gRNA efficacies by coprocessing screens that use the same gRNA library

The JACKS model provides estimates of gRNA efficacies, which match intuition for individual genes. For example, the smaller changes in representation for *KRAS* gRNAs 1 and 6 over the course of multiple cancer cell line screens (Fig. 1A) are appropriately captured in the differences of the posterior distributions. The estimates are concordant with both screen data derived efficacies from CERES (Meyers et al. 2017), as well as Doench–Root scores calculated from gRNA features only (Fig. 1B; Supplemental Fig. S1; Doench et al. 2016). They are consistent across randomly selected batches of cell lines for gRNAs targeting essential genes defined by Hart et al. (2014) (“Hart essential genes”) and for Hart nonessential genes (Supplemental Fig. S2). This reproducibility is Cas9-dependent (Supplemental Figs. S3, S4), suggesting that it arises from a response to gRNA action, even for known nonessential genes. Together, these findings support the use of JACKS efficacy values for improved screen analysis.

### Jointly processing screens with JACKS improves gene essentiality estimates

The gene essentiality estimates from JACKS measure the gene knockout's log_2_ fold change in frequency between control and treatment conditions, corrected for noise and gRNA efficacy. For example, as expected, knocking out the *KRAS* gene was inferred to have a greater impact on growth in cell lines known to harbor *KRAS* driver mutations in the Aguirre data set (Fig. 1A; Aguirre et al. 2016).

To examine JACKS’ ability to identify screen hits, we ranked genes by their essentiality and evaluated how well this discriminates Hart essential and nonessential genes. We first measured performance using the 0.2 partial area under the curve (“Ranking accuracy”; Methods) (Supplemental Fig. S5) and above the curve (“Ranking error”) metrics; equivalent results were obtained using alternative thresholds and criteria (Supplemental Figs. S6, S9; Supplemental Table S1). Increasing the number of experiments processed by JACKS from one to all available screens in each data set reduced the median ranking error by 10%, 10%, 23%, 26%, and 11%, respectively (Fig. 1C), with the first five to 10 lines providing the majority of the gains. Improvements were largest for the GeCKOv2 and TKOv1 libraries, likely due to the lower starting performance of those screens and more variable gRNA efficacy (Doench et al. 2016; Meyers et al. 2017). Similar results were obtained using an updated set of essential genes (Supplemental Fig. S10).

### JACKS outperforms existing single-screen methods

**Figure 2.**
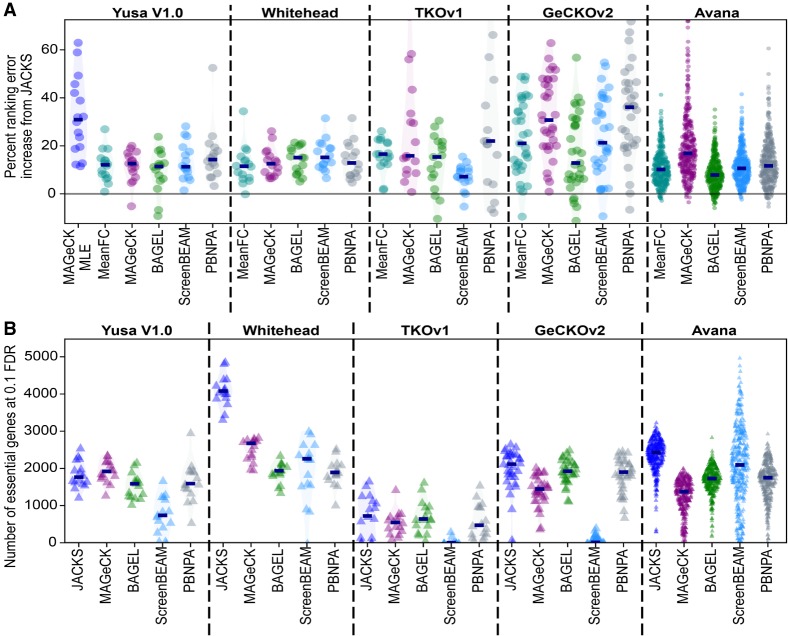
JACKS outperforms existing approaches. (*A*) JACKS outperforms existing alternatives at distinguishing essential genes. Percentage of ranking error increase (*y*-axis) compared to JACKS for five to six alternative analysis methods (*x*-axis) on five different libraries (panels). Every marker represents one cell line or time point sample; median increase is marked with a dark blue line segment, and estimated distributions are shaded. (*B*) JACKS identifies more essential genes compared with existing methods. Number of essential genes identified at a 0.1 false-discovery rate (*y*-axis) for JACKS and alternative analysis methods (*x*-axis). Every marker represents one cell line or time point sample.

We next compared the performance of JACKS to existing single-screen analysis methods. We considered averaging the log_2_ fold changes of all gRNAs targeting the gene (“MeanFC”), MAGeCK (Li et al. 2014), BAGEL (Hart and Moffat 2016), ScreenBEAM (Yu et al. 2016), and PBNPA (Jia et al. 2017). JACKS improved accuracy for 97%, 99%, 91%, 98%, and 98% of all cell lines tested, respectively, with a 12%, 21%, 9%, 13%, and 16% lower error on average (Fig. 2A). When applied to data from each cell line separately, the results for JACKS were comparable to the alternatives (Supplemental Fig. S11). This shows that although JACKS was designed to efficiently integrate information across experiments, there is no downside to using it on a single screen.

JACKS computes the magnitude of a gene's essentiality. To derive a *P*-value for significance testing, we use an empirically derived null distribution based on essentiality scores in a known set of negative control genes (Methods). A suitable threshold for calling hits can then be selected, for example, by controlling the false-discovery rate (Benjamini and Hochberg 1995). This is similar to the approach taken by BAGEL, except that we rely only on the provision of a negative control set rather than both negative and positive controls, as the latter is often more difficult to provide in practice. Using this method, we identify more essential genes than MAGeCK, BAGEL, ScreenBEAM, and PBNPA in 96%, 92%, 73%, and 90% of screens tested, respectively (Fig. 2B). Without ground truth, it is difficult to prove that these additional hits are real. However, we note that the number of findings is strongly correlated with a metric of screen quality (Spearman's *R* = 0.81 for JACKS, vs. 0.71, 0.60, 0.54, and 0.60 for MAGeCK, BAGEL, ScreenBEAM, and PBNPA, for number of hits vs. MeanFC ranking accuracy) (Supplemental Fig. S12), suggesting that JACKS is extracting additional signal in cases in which such signal is more likely to be present. Overall, all examined data sets benefited from joint screen analysis for accurate identification of essential genes.

### JACKS assumes common gRNA efficacies but not common gene essentialities across screens

**Figure 3.**
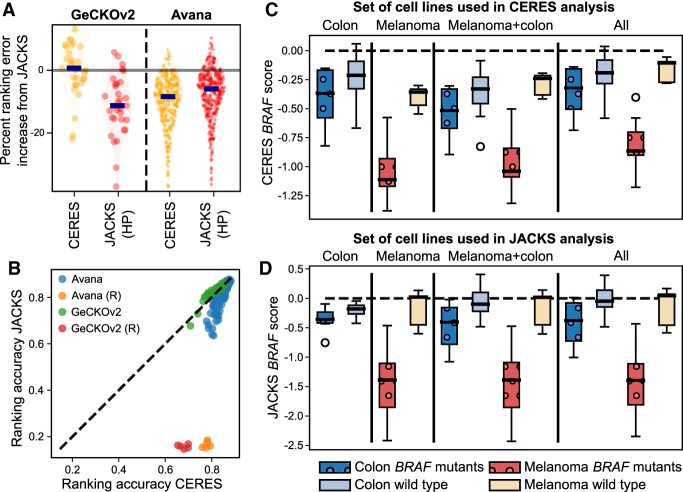
Assuming similar gene essentiality across experiments biases results. (*A*) Methods that assume similar gene essentialities across cell lines perform favorably compared to JACKS. Percentage of ranking error increase compared to JACKS (*y*-axis) for CERES (yellow) and JACKS with a hierarchical prior (HP) (red) for the GeCKOv2 and Avana libraries. Markers and shading as in Figure 2A. (*B*) CERES identifies essential genes from random data. Ranking accuracy of CERES (*x*-axis) compared to JACKS (*y*-axis) on cell lines (individual markers) from the Avana (blue) and GeCKOv2 (green) libraries, as well as five randomized experiments (yellow and red markers) included for comparison. Dashed line, *y* = *x*. (*C*) CERES’ preference for a common gene response across cell lines results in more similar scores for differentially essential genes, whereas (*D*) JACKS maintains differential signal between cell lines. CERES (*C*) and JACKS (*D*) gene essentiality scores for the *BRAF* gene in melanoma and colon cancer cell lines (colors) when processed with selections of cell lines (panels) from the Avana data set, grouped by *BRAF* mutation status (shading and patterns).

Two existing methods jointly model outputs from multiple screens: MAGeCK-MLE (Li et al. 2015) and CERES (Meyers et al. 2017). We could run MAGeCK-MLE on only the smallest, Yusa v1.0 data set because of large CPU and memory requirements (81 CPU days on four to 23 cores, 75 GB of RAM for 16 screens), and observed it to offer no improvement over standard MAGeCK (Fig. 2A). CERES performed equivalently to JACKS on GeCKOv2 data and was more accurate on the Avana data set (0.7% higher and 8.4% lower median ranking error, respectively) (Fig. 3A).

The CERES model assumes that gene essentiality signals are not independent across experiments. To evaluate the impact of this assumption, we introduced five additional screens into both the GeCKOv2 and the Avana data sets, each containing shuffled gRNA responses from a randomly selected cell line. CERES was able to identify essential genes from the randomized data with high accuracy, whereas JACKS achieved the expected near-random performance (Fig. 3B). We supplemented JACKS with an option to make a similar assumption of shared gene essentiality (JACKS [HP], Methods), and confirmed that this change resulted in comparable error to CERES on both GeCKOv2 and Avana data (Fig. 3A) and correspondingly improved the ability to extract signal from the lines with shuffled data (Supplemental Fig. S13).

Sharing gene effects across screens is beneficial for finding universal hits but could mask true context-specific signal. To test this possibility, we examined *BRAF* essentiality in melanoma, in which *BRAF* mutations are predictive of sensitivity to BRAF inhibitors (Chapman et al. 2011), and in colon cancer, in which *BRAF* mutations are less prevalent and predict only a weak response to BRAF inhibitors (Prahallad et al. 2012). Accordingly, JACKS’ estimated *BRAF* essentiality in the Avana data set is large in *BRAF* mutant melanoma lines, weak in *BRAF*-mutant colon cancer lines, and negligible in most other lines (median, −1.39 vs. −0.35 vs. 0.03) (Fig. 3D), regardless of the data set used in estimation. CERES’ preference for a common gene response alters its estimates depending on which other lines are selected for coprocessing. The *BRAF* essentiality score is lower in mutant melanoma lines when processed with all lines compared to when processed with melanoma lines alone (median, −0.86 vs. −1.11). Conversely, its essentiality is estimated to be larger in other lines when processed together with mutant melanoma lines instead of all data (median, −0.36 vs. −0.11, −0.52 vs. −0.32 and −0.33 vs. −0.19 for nonmutant melanoma lines, nonmutant colon lines, and mutant colon lines, respectively) (Fig. 3C). Including melanoma lines with colon cancer lines in JACKS estimation increases the separation of *BRAF* mutants from nonmutants in the colon cancer lines (AUC 0.72 vs. 0.78 in colon only and in colon + melanoma lines), suggesting that *BRAF* mutation status is still predictive in colon cancer, if only of a much weaker response.

### JACKS allows reduced experiment sizes

**Figure 4.**
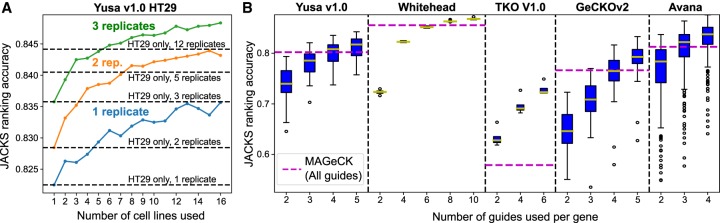
JACKS enables reduced screen size and cost. (*A*) Average JACKS ranking accuracy (*y*-axis) on HT29 cell line for increasing numbers of coprocessed cell lines (*x*-axis), and different number of technical replicates (colors). Two hundred cell lines were randomly sampled for each point on the graph and results averaged. As a reference, the same metric is plotted in increasing numbers of HT29 replicates (*y*-axis) processed by JACKS without the other cell lines (dashed lines). (*B*) JACKS ranking accuracy (*y*-axis) for increasing numbers of gRNAs (*x*-axis) from five different libraries (panels) using two replicates per cell line, compared to MAGeCK used on all five gRNAs and all available (two to four per cell line) replicates (dashed line). Box plot as in Figure 1C.

Finally, we tested if improved analysis methods can be used to reduce experiment size without compromising findings. First, we considered the number of replicate screens. We performed 12 replicate experiments with the Yusa v1.0 library on the HT29 cell line (Methods) and combined these with the above-described Yusa v1.0 data set, which contains two to three replicates each for 15 additional cell lines. Identifying essential genes from three replicates of HT29 using JACKS, when coprocessing with three replicates from each of the other 15 lines, outperformed processing of the 12 HT29 replicates in isolation. Similarly, applying JACKS to just two replicates from HT29 and each of the other lines outperformed analysis of five HT29 replicates (Fig. 4A). We then considered whether the gRNA numbers could similarly be reduced without sacrificing performance and evaluated the accuracy of JACKS with specific numbers (two to 10) of randomly picked gRNAs for each gene in each of the five libraries. Although performance decreased with reduction of gRNA numbers, just three randomly selected Avana gRNAs for each gene and two replicates were enough to outperform MAGeCK with three replicates and all five gRNAs (Fig. 4B). Combined, using two replicates and three gRNAs, would reduce the required experiment size 2.5-fold, directly impacting the scale and cost of screens.

## Discussion

We presented JACKS, a Bayesian model for joint analysis of screens performed with the same gRNA library. We showed that JACKS improves identification of hits in a single screen by using information from all the available experiments. As a result, JACKS outperforms existing analysis methods in the vast majority of lines tested across five different data sets and gRNA libraries and does so without sacrificing context-specific signal. This allows greatly reduced experiment sizes while retaining performance equivalent to MAGeCK, the current analysis standard. Ability to carry out screens with smaller libraries and to efficiently analyze them is especially important for some of the most interesting applications, such as mapping genes in primary cells that can only be obtained in limited numbers and propagated for a short time, or in genetic interaction screens, in which controlling the scale of experiments is one of the central issues.

The parameters inferred by JACKS are useful for interpreting screen results. We found that the estimated gRNA efficacies were reproducible across cell lines both for nominally essential and for nonessential genes. This indicates that effects of even small magnitude, if consistent across many experiments, can be used for inference. The posterior distribution of a gene's essentiality can be used to derive a point estimate of knockout effect in the screen or also to assign a probability of positive or negative effect. However, we found that ranking genes by probability gives poorer performance (Supplemental Fig. S14) than ranking by effect size as it unduly favors genes with effects that are reproducibly different from zero, but only small in size.

Copy number variation has been shown to play a role for essential gene inference from CRISPR/Cas9 screens (Aguirre et al. 2016; Meyers et al. 2017; Iorio et al. 2018; Wu et al. 2018) and is modeled in CERES. Although JACKS does not account for this signal distortion, the experiments described here should be independent of copy number effects. JACKS can be used in combination with CRISRPcleanR (Iorio et al. 2018) or other preprocessing methods that remove these effects.

Recompiling published data sets and rerunning a full analysis for each new screen may be prohibitive for many in practice. We have precomputed gRNA efficacies for these existing libraries, which can be used to process a single screen to achieve equivalent performance to the full JACKS model (Pearson's *r*^2^ > 0.99) (Supplemental Fig. S15). These are available at https://github.com/felicityallen/JACKS/tree/master/reference_grna_efficacies.

## Methods

### Joint analysis of CRISPR-Cas9 knockout screens (JACKS)

We define the observed log_2_ fold change of the *i*th guide targeting the *g*th gene in the *l*th treatment condition as *y*_*g,i,l*_, where this value is computed as the mean across median-normalized replicate measurements as follows:

$$y_{g,i,l}=\frac{1}{R_{T}}\overset{R_{T}}{\underset{r=1}{\sum }}T_{g,i,l,r}-\frac{1}{R_{C}}\overset{R_{C}}{\underset{r=1}{\sum }}C_{g,i,r}.$$

Here, *T*_*g*,*i*,*l*,*r*_ = log_2_(*t*_*g*,*i*,*l*,*r*_ + 32) − median(*T*_:,:,*l*,*r*_) and *C*_*g*,*i*,*r*_ = log_2_(*c*_*g*,*i*,*r*_ + 32) − median(*C*_:,:,*r*_) are log-transformations of the raw read counts *t*_*g,i,l,r*_ and *c*_*g,i,r*_ for the *r*th replicates in the treatment and control samples, respectively, and the median functions operate over all guides across all genes in each respective replicate; *R*_*T*_ and *R*_*C*_ are the number of replicates in those respective samples. The pseudocount of 32 is added as a prior that prevents strong signals due to low library representation. This is a softer alternative to the more common practice (e.g., Hart et al. 2017) of removing all gRNAs with less than 30 reads.

We model *y*_*g,i,l*_ as a Gaussian distribution

$$P(y_{g,i,l}|x_{g,i},w_{g,l},\tau_{g,i,l})=N(y_{g,i,l};\;x_{g,i}w_{g,l},\;\tau_{g,i,l}^{-1}),$$

where

- $w_{g,l}∼N(\mu_{w},\sigma_{w}^{2})$ is the condition-dependent gene effect of the *g*th gene in the *l*th treatment condition, where *μ*_*w*_ and $\sigma_{w}^{2}$ are set to 0 and 1000, respectively, reflecting a weak prior (average change, 0; standard deviation of change over 32 in log_2_ scale) that is constant across conditions.
- $x_{g,i}∼N(\mu_{x},\sigma_{x}^{2})$ is the condition-independent gRNA efficacy of the *i*th guide targeting the *g*th gene. A stronger prior is specified, with $\mu_{x}=\sigma_{x}^{2}=1$ to reflect the prior belief that most gRNAs work moderately well, as well as to prevent overfitting. Scaling *x*_*g,i*_ up by a constant factor and *W*_*g,l*_ down by the same factor results in an identical optimization. So to make the model identifiable, the means of the approximate posteriors of *x* are normalized during inference within each gene, such that their median-emphasized average is one, according to
  $$\frac{1}{N_{g}}\left(\overset{N_{g}}{\underset{i=1}{\sum }}E\left(x_{g,i}\right)+\mathrm{median}\left(E\left(x_{g,:}\right)\right)-\frac{1}{2}\max\left(E\left(x_{g,:}\right)\right)-\frac{1}{2}\min\left(E\left(x_{g,:}\right)\right)\right)=1,$$
  where *N*_*g*_ is the number of gRNAs targeting gene *g*, and *x*_*g*_,: refers to all efficacies for gene *g*. This median-emphasized average is intended to select an appropriate reference point for *w* that accounts for all observations for each gene but up-weights the median and down-weights the extremes.
- *τ*_*g*,*i*,*l*_ ∼ Γ(*a*_*g*,*i*,*l*_, *b*_*g*,*i*,*l*_) is the precision of *y*_*g,i,l*_, which uses a nonparametric approach to assign an empirical Bayes prior that accounts for the mean-dependent variability of the log_2_ count values within the replicate measurements of both the treatments and controls. This provides a data-driven and computationally feasible alternative to the parametric approach of modeling counts using a negative binomial distribution, as used in MAGeCK (Li et al. 2014). Given that, in general, only two to four replicate screens are performed, direct empirical estimates of these variances are poor. Consequently, we instead compute a smoothed mean-dependent estimate of this empirical variance based on all gRNAs in each condition and then assign the priors on *τ*_*g,i,l*_ as follows:
  1. Compute the mean and variance over replicates for all median-normalized log counts in each treatment and control sample, that is, the means and variances of *T*_*g,i,l,*:_ and *C*_*g,i,*:_, where “:” in the subtexts denotes all replicate measurements.
  2. Sort these mean–variance pairs by their mean value.
  3. Apply a simple moving average filter to the variance values such that each estimated variance becomes a mean of the empirical variances of either (800 gRNAs or 1% of the total number of gRNAs, whichever is smaller) with closest mean in that cell line (or control), with an additional correction that ensures monotonicity in the relationship (scanning from highest mean; any steps lower are held constant). Denote these estimated variances for each treatment and control measurement as ${\hat{\sigma }}_{T,g,i,l}^{2}$ and ${\hat{\sigma }}_{C,g,i}^{2}$, respectively.
  4. Assign the prior parameters for τ_*g*,*i*,*l*_, *a*_*g*,*i*,*l*_ = *κ* and $b_{g,i,l}=\kappa ({\hat{\sigma }}_{T,g,i,l}^{2}+{\hat{\sigma }}_{C,g,i}^{2})$, where *κ* determines the strength of the prior (we used *κ* = 0.5), which assigns an expected variance of *y*_*g,i,l*_ as ${\hat{\sigma }}_{T,g,i,l}^{2}+{\hat{\sigma }}_{C,g,i}^{2}$, the sum of the estimated treatment and control variances.

Variational inference is used to infer the posterior distributions of *x*, *w*, and *τ*. The closed form update equations for the posterior distributions of each variable are

$$\begin{array}{rl} & Q_{x_{g,i}}∼N\left(\frac{\frac{\mu_{x}}{\sigma_{x}^{2}}+\sum_{l}\tau_{g,i,l}^{*}y_{g,i,l}E_{w}[w_{g,l}]}{\frac{1}{\sigma_{x}^{2}}+\sum_{l}\tau_{g,i,l}^{*}E_{w}[w_{g,l}^{2}]},\frac{1}{\frac{1}{\sigma_{x}^{2}}+\sum_{l}\tau_{g,i,l}^{*}E_{w}[w_{g,l}^{2}]}\right),\\ & Q_{w_{g,l}}∼N\left(\frac{\frac{\mu_{w}}{\sigma_{w}^{2}}+\sum_{l}\tau_{g,i,l}^{*}y_{g,i,l}E_{x}[x_{g,i}]}{\frac{1}{\sigma_{w}^{2}}+\sum_{l}\tau_{g,i,l}^{*}E_{x}[x_{g,i}^{2}]},\frac{1}{\frac{1}{\sigma_{w}^{2}}+\sum_{l}\tau_{g,i,l}^{*}E_{x}[x_{g,i}^{2}]}\right),\\ & Q_{\tau_{g,i,l}}∼\Gamma (a_{g,i,l}+0.5,b_{g,i,l}+0.5\beta_{g,i,l}^{*}), \end{array}$$

where

$$\begin{array}{rl} & \beta_{g,i,l}^{*}=E_{\overset{-}{\tau }}[{\left(y_{g,i,l}-x_{g,i}w_{g,l}\right)}^{2}]=y_{g,i,l}^{2}-2y_{g,i,l}E_{x}[x_{g,i}]E_{w}[w_{g,l}]+E_{x}[x_{g,i}^{2}]E_{w}[w_{g,l}^{2}],\\ & \tau_{g,i,l}^{*}=E_{\tau }[\tau_{g,i,l}]=\frac{a_{N,{\hat{\mu }}_{g,l}}+0.5}{b_{N,{\hat{\mu }}_{g,i,l}}+0.5\beta_{g,i,l}^{*}}. \end{array}$$

To determine *P*-values, JACKS requires a set of negative control genes or gRNAs. These can be known nonessential genes (in the context of growth screens) or control guides known to cut only in unimportant genomic regions (e.g., noncoding regions). We compute *P*-values for all treatment conditions simultaneously by:

1. Randomly selecting any gene from the library used to run the screen, and setting *N* to be the number of gRNAs targeting this gene in the library.
2. Randomly selecting *N* gRNAs from the full set of negative controls, call this set of gRNAs a negative pseudogene.
3. Repeat steps 1 and 2 to generate 2000 negative pseudogenes.
4. Run JACKS inference to compute $\hat{w_{g,l}}E[w_{g,l}]$ values for each of these pseudogenes (from the normalized per-gRNA fold changes already computed within JACKS).
5. For each individual treatment condition, compute a nonparametric distribution Φ_*l*_(*w*_*g*,*l*_) over the 2000 $\hat{w_{g,l}}$ values using the gaussian kde function from scipy.stats (http://www.scipy.org/, accessed December 12, 2018).
6. For each $\hat{w_{g,l}}$ value in the full JACKS results of all genes, the *P*-value is ${\mathrm{Pr}}_{\Phi_{l}}(w_{g,l}<\hat{w_{g,l}})$ and is computed numerically using the integrate_box_1d method within gaussian_kde.

The resampling of gRNAs from the control set makes the method more robust to mislabeled control genes. For example, Hart nonessential genes were originally defined by a lack of measured RNA expression (Hart et al. 2014); however, a small proportion of these gRNAs show a strong growth effect in some screens, which distorts the null distribution. Resampling the gRNAs spreads these errors across genes such that their effect on any one gene is less pronounced.

### Classification of Hart essential genes on pooled knockout screens

The five genome-wide pooled CRSIPR/Cas9 knockout screen data sets used here were compiled from data in Koike-Yusa et al. (2014), Hart et al. (2015), Wang et al. (2015, 2017), Aguirre et al. (2016), Meyers et al. (2017), and Iorio et al. (2018). The compiled sets are listed on figshare (see Data Access) with complete instructions for recompilation in the respective README files. We used the core essential genes and nonessential gene sets defined by Hart et al. (2014), using siRNA and expression data, restricted to those that were targeted by guides within each library. Although an updated set of essential genes was defined in Hart et al. (2017), these definitions use data from the Yusa v1.0, Whitehead, and TKO data sets. To avoid circularity, we could therefore only use this set to assess performance on the GeCKO and Avana data sets and saw similarly improved performance over other methods with this set (Supplemental Fig. S10).

We evaluated performance using the 0.2 partial area under the curve (0.2 pAUC; “ranking accuracy”) and equivalent above the curve (0.2 pAAC; “ranking error”) metrics (Fig. 2A; Supplemental Fig. S3). AUCSs are robust measures commonly used to assess the ability of a method to distinguish between two categories; the partial aspect focuses this metric on the more relevant part of the curve where the false-positive rate is below 20%. Equivalent results were obtained using other thresholds (0.1 pAUC, full AUC) and performance criteria (recall at fixed false-discovery rate, false-positive rate at fixed recall, delta AUC [essential vs. all genes AUC − nonessential vs. all genes AUC]) (Supplemental Figs. S6–S9; Supplemental Table S1). All metrics were calculated directly from the receiver operator curve returned by the roc function in scikit-learn (Pedregosa et al. 2011) applied to the estimated gene essentiality measures.

### Comparisons with other methods

Scripts used to run all other methods are available on GitHub and in the Supplemental Material (see Data access). Input formats for each method were inconsistent, and so the data were reformatted for compatibility. MeanFC was computed using a custom script that computed the mean median-normalized log_2_ fold changes across replicates for each gRNA as done in JACKS (described above) and then assigned each gene a score equal to the mean of this value across all gRNAs targeting that gene. The MAGeCK (Li et al. 2014) v0.5.7 test command was used to run MAGeCK, and the mle command was used to run MAGeCK MLE (Wu et al. 2018). BAGEL (Hart and Moffat 2016) v0.91 was slightly modified to take a mean of the control samples (when multiples were available) before computing the fold changes, because BAGEL otherwise expects a single control measurement. ScreenBEAM 1.0.0 was run in R 3.4.0 (R Core Team 2018) using all recommended defaults for NGS processing. Ranking accuracy results use the B output, whereas sets of essential genes were determined using the *P*-value output. PBNPA 0.0.3 was run using R 3.4.0 (R Core Team 2018) with all recommended defaults, except that the *P*-value and FDR thresholds were set to 10.0 and 100.0, respectively, to deactivate them, so that all genes results were recorded. The negative *P*-value outputs were used for all presented results. CERES v0.0.0.9 was run with λ_*g*_ = 0.561 for Avana and λ_*g*_ = 0.681 for GeCKOv2 as recommended in Meyers et al. (2017). We note that in CERES, λ_*g*_ controls the extent to which common gene responses across cell lines are favored, and so altering this value would alter the results presented here. However, as deciding a correct value for this parameter without overfitting to the test at hand is nontrivial, we relied on the published recommended values selected for these same data sets and did not attempt to optimize this further. We believe this is representative of general usage of this program in the absence of alternative guidance, but note that this may be a worthwhile area for future exploration.

### Concordance and reproducibility of gRNA efficacy values

We used the Rule-Set 2 scores from Doench et al. (2016) (“Doench–Root scores”), which provide a sequence-based prediction of gRNA efficacy. Concordance between these scores and JACKS gRNA efficacy estimates was assessed similarly to the method previously described by Meyers et al. (2017), by binning gRNAs by their Doench–Root scores and then looking for increased fractional representation of those gRNAs deemed to have higher *x* value in higher scoring Doench–Root bins. Unlike in Meyers et al. (2017), we restricted this analysis to gRNAs targeting Hart essential genes as we did not expect JACKS (or CERES) to derive any meaningful gRNA efficacy information from genes with no screen activity.

Reproducibility estimates for JACKS gRNA efficacy values were obtained by running JACKS on the Avana batch 0 and batch 1 data sets separately. We computed JACKS gRNA efficacy estimates (𝔼[*x*]), for those gRNAs targeting Hart essential genes, on 100 randomly selected sets of *N* cell lines from each batch, where *N* = 1, 3, 5, 8, 10, 15, 20, 25, and 30. We then computed the Spearman's correlation coefficient between the two estimates for each set to obtain a distribution of correlations.

### Additional Yusa v1.0 screens

Previously unpublished screens using the Yusa v1.0 library in HT29, CO205, HuPT4, SW1990, A375 (Supplemental Table S2), and an additional HT29 line without Cas9 (Supplemental Table S3), were performed using the same screening protocol as in Iorio et al. (2018).

### Construction of random line data

To generate the five randomly shuffled cell lines for the GeCKOv2 (Supplemental Table S4) and Avana (Supplemental Table S5) libraries, we randomly selected three replicates for each line from existing replicates in other lines. For each of those replicates, we computed the log_2_ median-normalized fold changes as in JACKS method and then randomly shuffled those fold changes across all guides. The fold changes were then converted back to raw counts, accounting for the control values of their reassigned gRNAs. The script used to create these lines is included as Supplemental Code.

### JACKS with hierarchical prior (HP)

To create a version of JACKS that favors similar gene essentialities across cell lines, we added a hierarchical prior (HP), setting the prior mean *μ*_*w*_ and variance $\sigma_{w}^{2}$ on *w*_*g,l*_ to the current estimated mean of 𝔼[*w*_*g*,*l*_] across all cell lines and to three times the current estimated variance of 𝔼[*w*_*g*,*l*_], respectively, at each update step in the variational inference. This encourages each *w*_*g,l*_ to be more similar to that in the other lines, with the effect being stronger when there is a more consistent response across lines.

### Compilation of *BRAF* mutant and wild-type cell lines

*BRAF* mutation status for cell lines in the Avana data set were obtained from the Cancer Cell Line Encyclopedia data portal (Barretina et al. 2012). Cell lines selected for the melanoma and colon cancer sets were those that were either *BRAF* wild-type or *BRAF* mutant but which did not have amplifications or deletions in the *BRAF* gene to avoid issues with copy number differences in comparisons between JACKS and CERES.

### Random sampling to assess the impact of number of cell lines, replicates, and gRNAs

To investigate the effect of increasing the number of cell lines coprocessed by JACKS (Fig. 1C) for a given cell line under test, we bootstrap sampled (with replacement) the requisite number of other cell lines, randomly selecting two replicates from each. We ran JACKS on each set sampled in this manner and recorded the gene scores for the cell line under test. We repeated this 200 times for each test cell line and condition, computing the average ranking accuracy (0.2 partial AUC score) across repetitions for each test cell line. The box plots in Figure 1C show the distribution of these mean scores across cell lines. The same procedure was used to assess the effect of the number of replicates in the Yusa v1.0 HT29 data (Fig. 3A), except that the test line was always HT29, and the number of replicates was also altered. This procedure was also used to assess the impact of reducing the number of gRNAs (Fig. 3B), except that the full set of cell lines was used in all samples, and the random sampling was instead taken (without replacement) on the available gRNAs for each gene.

## Data access

JACKS is available under an MIT license at www.github.com/felicityallen/JACKS in Python, with user documentation at https://github.com/felicityallen/JACKS/blob/master/jacks/README.md. Data and results of analysis from this study are available at https://www.doi.org/10.6084/m9.figshare.6002438 and as Supplemental Code.

## Supplementary Material

Supplemental Material
