# Supplementary material for: JACKS: joint analysis of CRISPR/Cas9 knockout screens
Source: Genome Res. 2019 Mar;29(3):464–71. doi: 10.1101/gr.238923.118 (PMC6396427; doi:10.1101/gr.238923.118)
Supplement: Supplemental Material [file supp_29_3_464__index.html]

Supplemental Material 

# JACKS: joint analysis of CRISPR/Cas9 knockout screens

## Supplemental Material

- Supplemental\_Code.zip
- Supplemental\_Table\_S2.txt
- Supplemental\_Table\_S3.txt
- Supplemental\_Table\_S4.csv
- Supplemental\_Table\_S5.csv
- Supplemental\_Table\_S1.xlsx
- Supplemental\_Figures.pdf
