# Supplementary material for: JACKS: joint analysis of CRISPR/Cas9 knockout screens
Source: Genome Res. 2019 Mar;29(3):464–71. doi: 10.1101/gr.238923.118 (PMC6396427; doi:10.1101/gr.238923.118)
Supplement: Supplemental Material [file supp_gr.238923.118_Supplemental_Figures.pdf]

Supplementary Figures

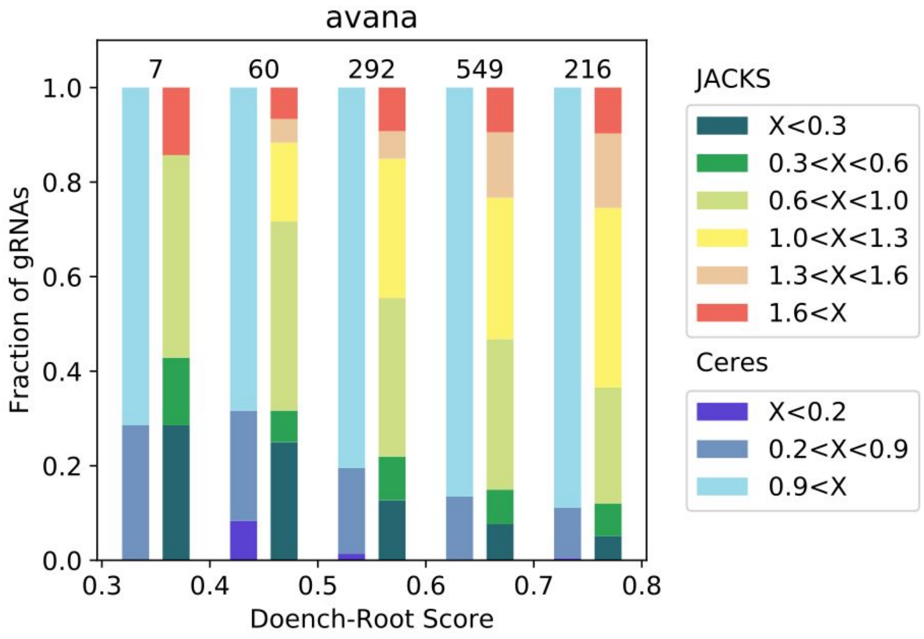

Figure S1: JACKS gRNA efficacy estimate is concordant with CERES estimate and Doench-Root score on the Avana library screens. Fraction of gRNAs (y-axis) in each of the Doench-Root score bins (x-axis) for different strata of CERES and JACKS inferred gRNA efficacies ("x", colors). Number of gRNAs in each column is marked above the bar.

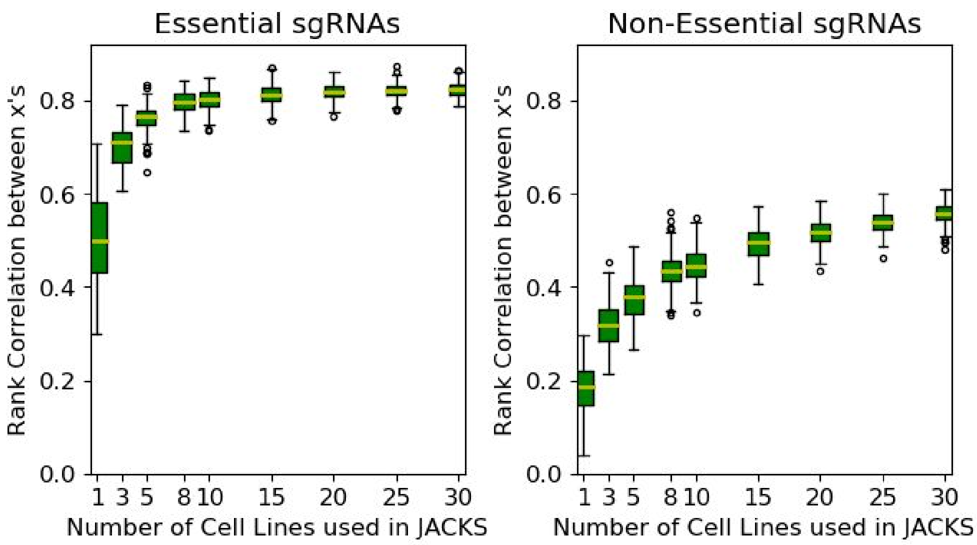

Figure S2: Estimates of gRNA efficacy are reproducible. Spearman's R (y-axis) for gRNA efficacy estimates from two mutually exclusive batches of Avana library experiments, restricted to gRNAs targeting only Hart essential genes (left) and Hart non-essential genes (right). For increasing number of cell lines used in JACKS (x-axes), 100 random sets of lines were picked for each batch, JACKS was run to infer posterior of gRNA efficacy \$x\$, and its expected value was used for calculating the correlation.

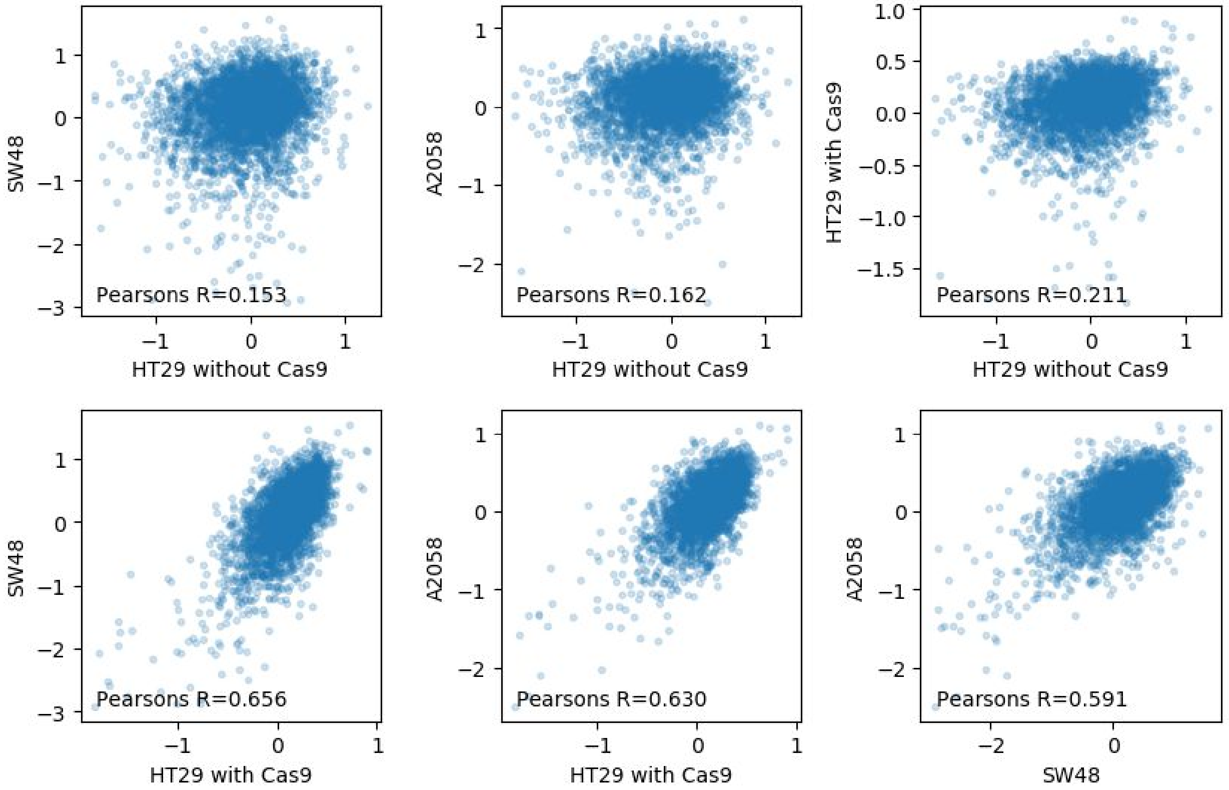

Figure S3: Log fold changes for gRNAs targeting non-essential genes are well correlated (>0.59) when Cas9 is present in the cells, but less so in cells without Cas9 (<0.21). Mean log fold change across replicates for gRNAs targeting Hart non-essential genes (markers; x,y axis) comparing a screen in HT29 line without Cas9 (top row panels) or with Cas9 (bottom row panels) to three other screens using the same library (panels x-axis), with Pearson's R reported. gRNAs were filtered to remove those with a mean fold change less than -2 in the "HT29 with Cas9" screen. The reproducibility of gRNA efficacies for non-essential genes for Cas9 positive lines, but lack of reproducibility in Cas9 negative line comparisons indicates that gRNA activity causes a reproducible response even for known non-essential genes, and suggests that reproducibility of gRNA effects is not due to use of a common control measurement, as this would be expected to also impact the screen without Cas9.

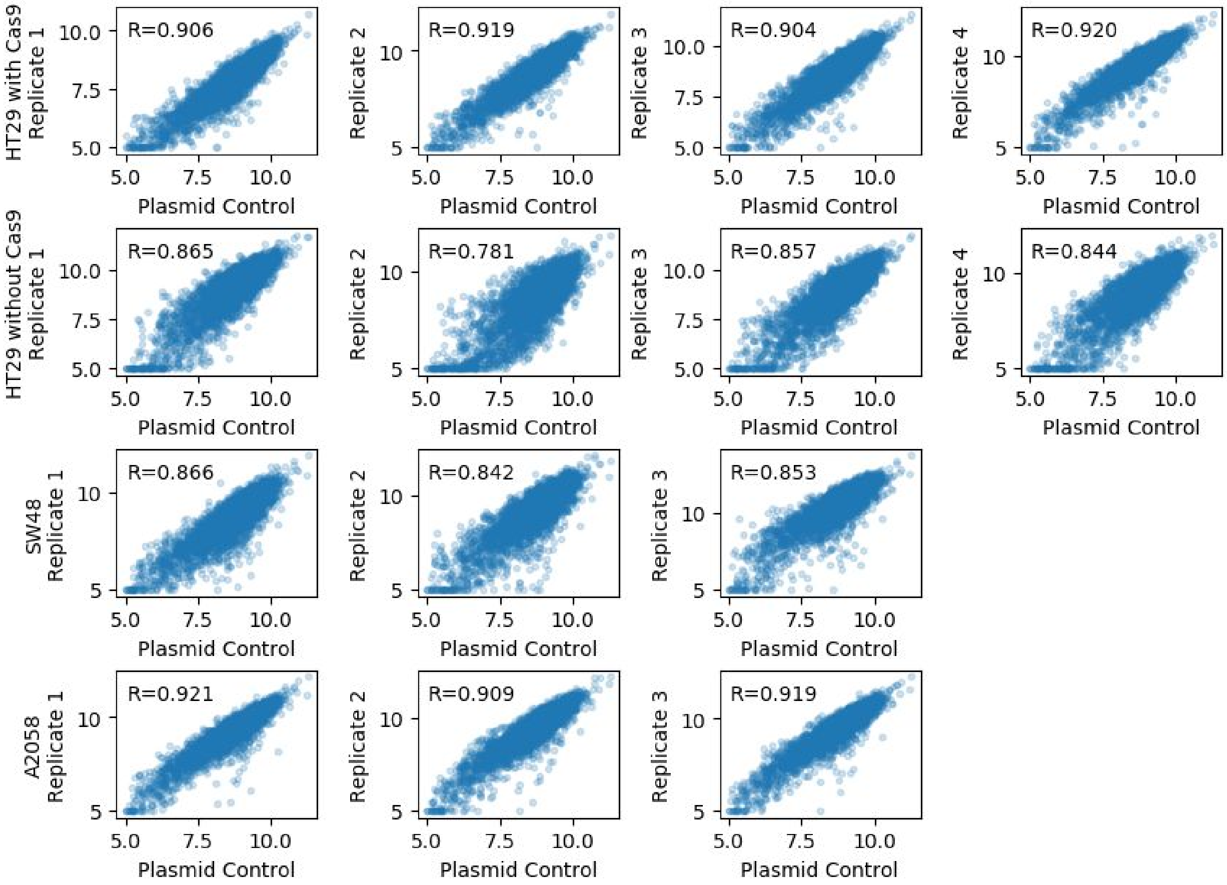

Figure S4: Log counts for gRNAs targeting Hart non-essential genes in screens used in Figure S3 are well correlated with plasmid controls. This test was used to check that the results in Figure S3 were not just due to poor screen quality in the lines without Cas9. Log2(count + 32) for gRNAs in individual replicates of each of the lines using in Figure S3 (y-axis) compared against their equivalent measures in the plasmid control (x-axis). Text gives Pearson's R of x vs y.

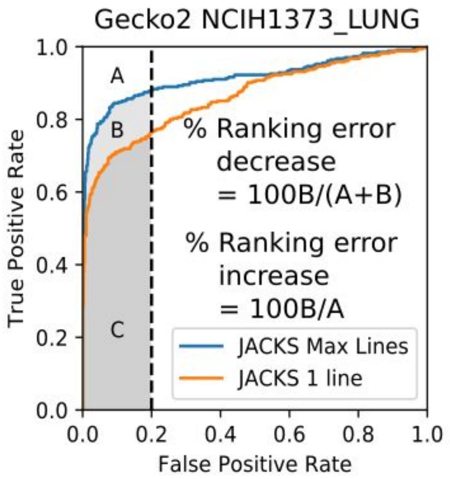

Figure S5: Calculating ranking error and ranking accuracy from receiver operator curve. True positive rate (y-axis) for fixed false positive rate (x-axis) for distinguishing true positive from true negative genes according to Hart (2014) using expected gene essentiality from JACKS run on single line (orange) or all 33 GeCKOv2 lines (blue). Dashed line denotes 0.2 false positive rate, ranking accuracy (area under the curve, grey), and ranking error (area above the curve, white) and their change are demonstrated for the NCIH1373_LUNG cell line.

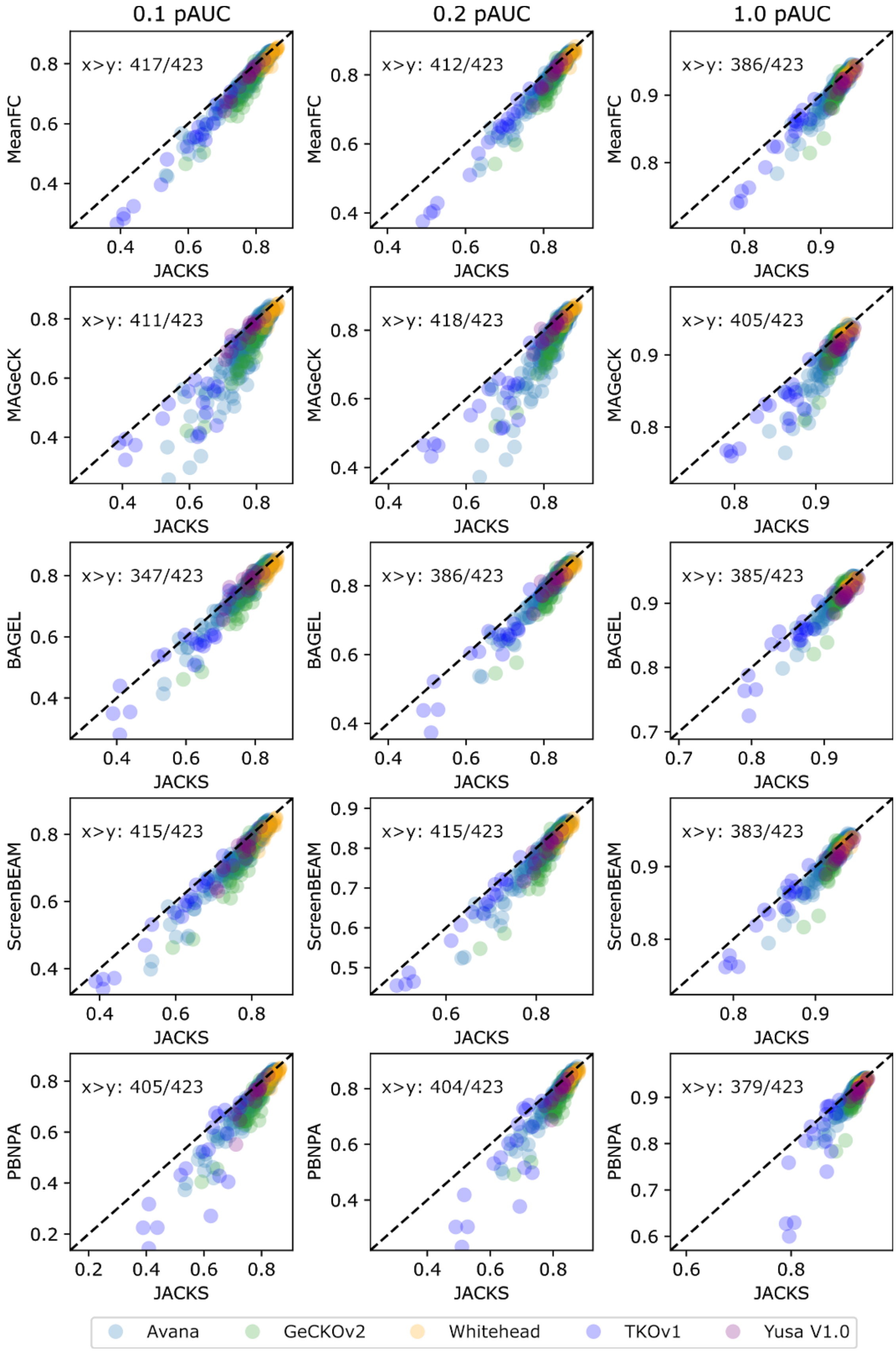

0.1 pAUC
0.2 pAUC
1.0 pAUC
x>y: 417/423
x>y: 412/423
x>y: 386/423
x>y: 411/423
x>y: 418/423
x>y: 405/423
x>y: 347/423
x>y: 386/423
x>y: 385/423
x>y: 415/423
x>y: 415/423
x>y: 383/423
x>y: 405/423
x>y: 404/423
x>y: 379/423
MeanFC
MAGeCK
BAGEL
ScreenBEAM
PBNPA
JACKS
Avana
GeCKOv2
Whitehead
TKOv1
Yusa V1.0

Figure S6: JACKS performs favourably compared to alternatives when measured on area under the curve metrics. JACKS run with all available lines (x-axis) compared to alternative methods (y-axis; first column - mean log2 fold change in gRNA representation; second column - MaGECK p-value; third column - BAGEL essentiality estimate) for cell lines (individual markers) from three different libraries (Avana - blue, GeCKOv2 - green, Yusa v1.0 - purple). First row: partial area under the curve at 0.1 FPR (0.1 pAUC); second row: at 0.2 FPR (0.2 pAUC); third row: at 1.0 FPR (AUC). Number of cell lines for which JACKS has higher pAUC than alternatives is denoted on the plot.

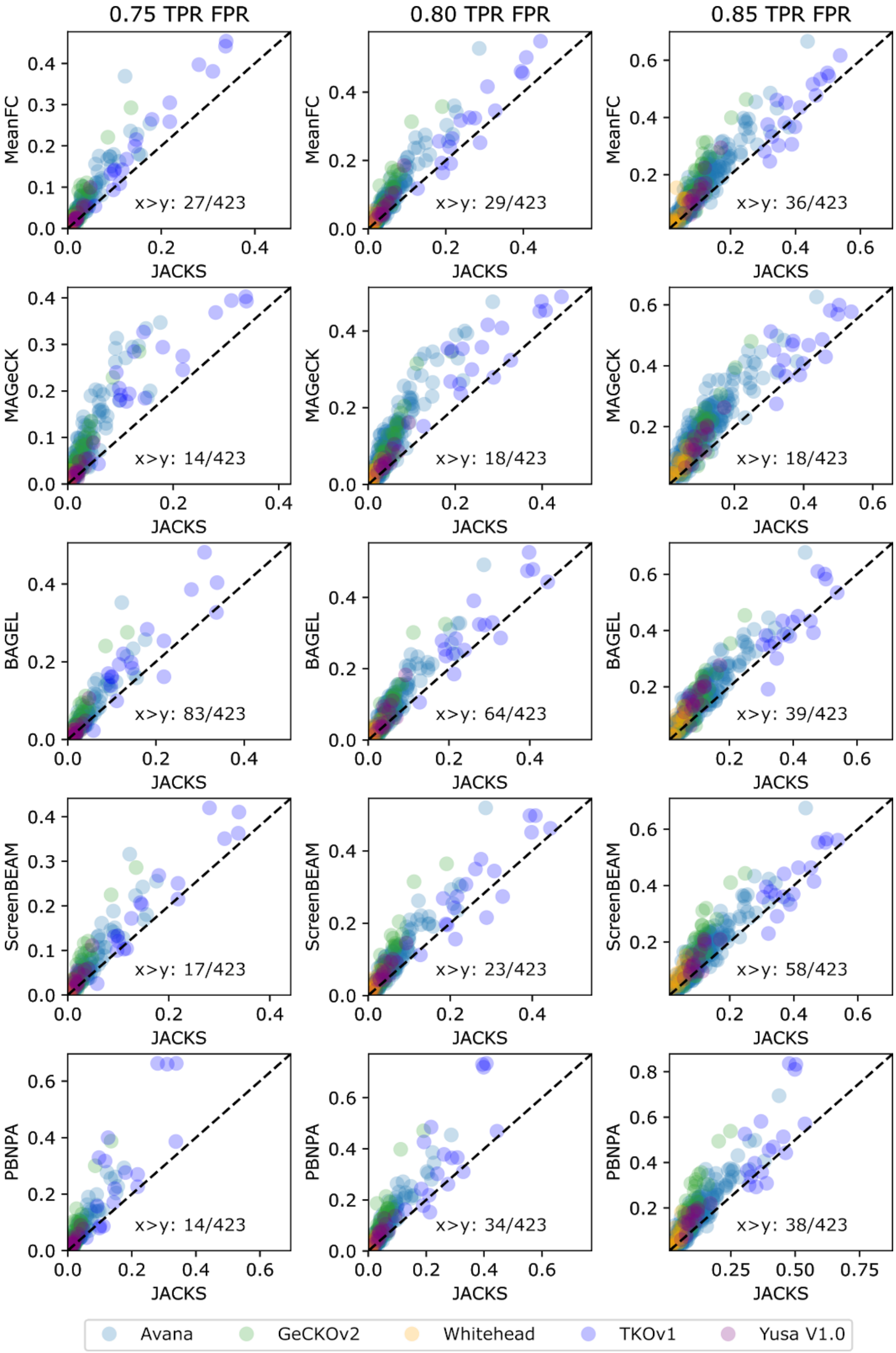
0.75 TPR FPR
0.80 TPR FPR
0.85 TPR FPR
MeanFC
MAGeCK
BAGEL
ScreenBEAM
PBNPA
JACKS
x>y: 27/423
x>y: 29/423
x>y: 36/423
x>y: 14/423
x>y: 18/423
x>y: 18/423
x>y: 83/423
x>y: 64/423
x>y: 39/423
x>y: 17/423
x>y: 23/423
x>y: 58/423
x>y: 14/423
x>y: 34/423
x>y: 38/423
Avana
GeCKOv2
Whitehead
TKOv1
Yusa V1.0

Figure S7: JACKS performs favourably compared to alternatives when measured by false positive rate at fixed recall. Axes, columns, colors as in Figure S6. First row: false positive rate at 0.75 true positive rate (TPR); second row: at 0.80 TPR; third row: at 0.85 TPR. Number of cell lines for which JACKS has higher false positive rate than alternatives is denoted on the plot.

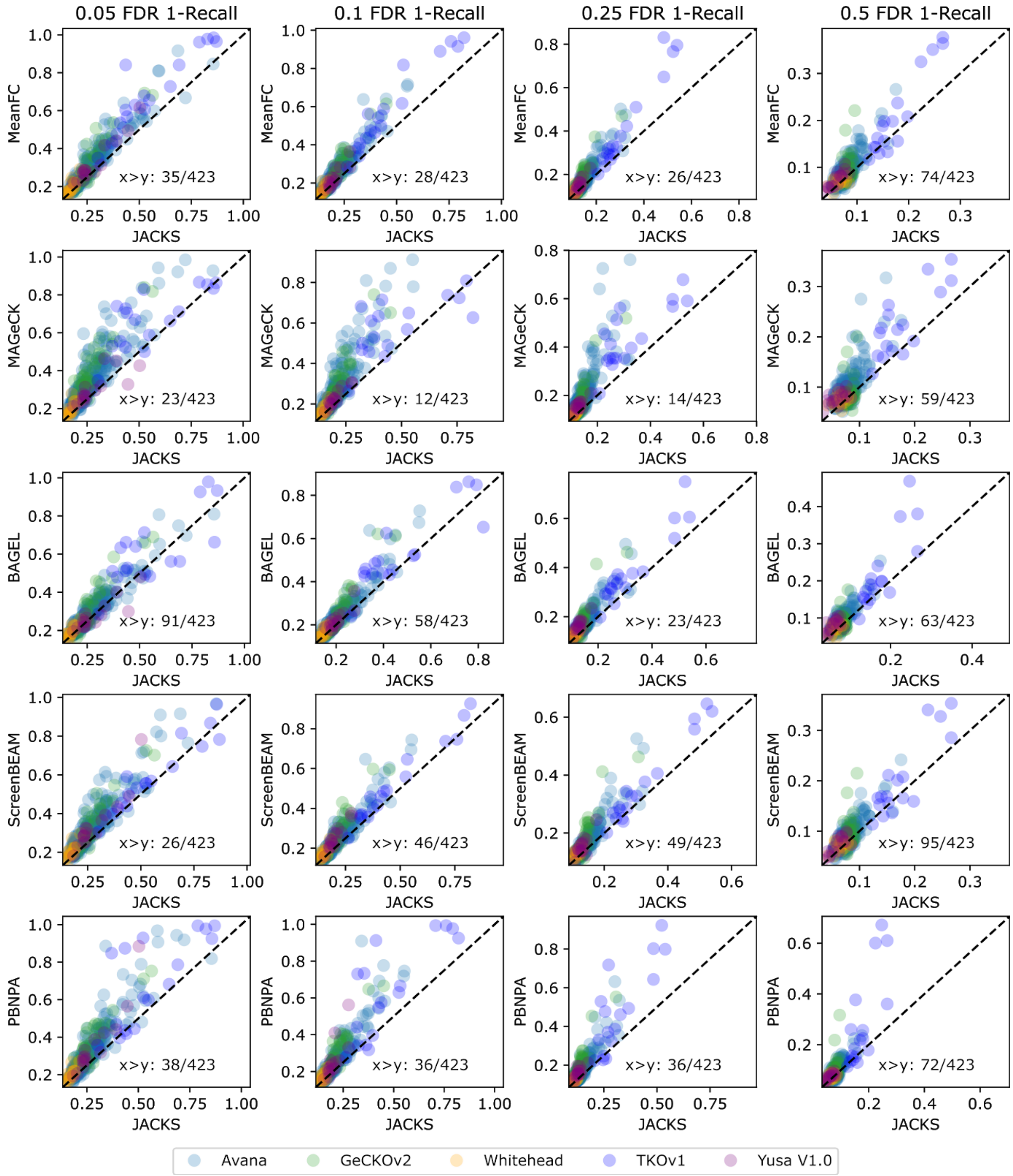

Figure S8: JACKS performs favourably compared to alternatives when measured by false negative rate at fixed false discovery rate. Axes, columns, colors as in Figure S6. First row: false negative rate at 0.05 false discovery rate (FDR); second row: 0.1 FDR; third row: 0.25 FDR; fourth row: 0.5 FDR. Number of cell lines for which JACKS has higher false negative rate than alternatives is denoted on the plot.

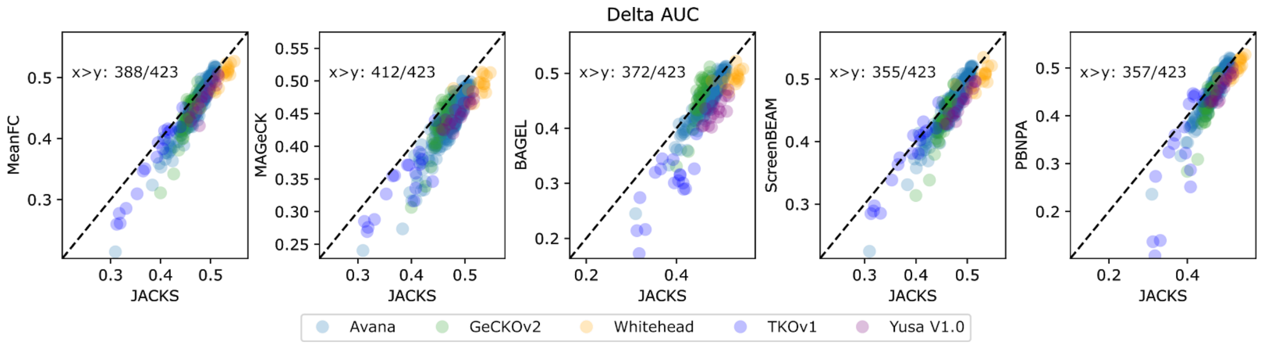

Figure S9: JACKS performs favourably compared to alternatives when measured by delta AUC. Axes, columns, colors as in Figure S6. Number of cell lines for which JACKS has better delta AUC than alternatives is denoted on the plot.

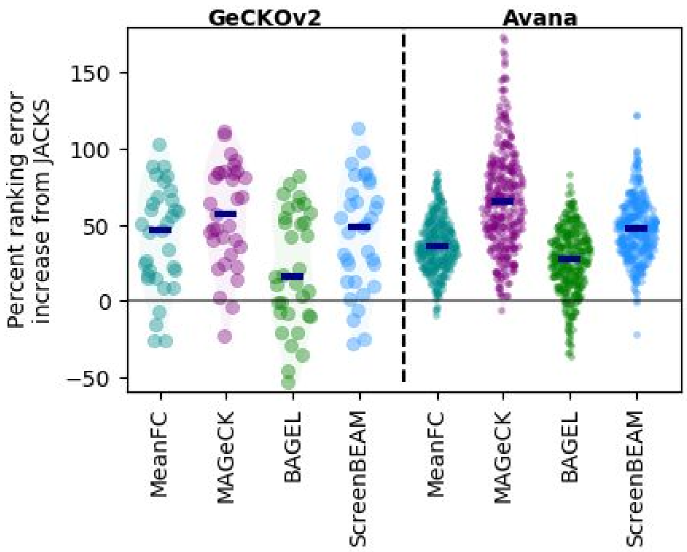

Figure S10: JACKS outperforms existing alternatives at distinguishing essential genes when using the Hart 2017 essential gene set. Percent ranking error increase (y-axis) compared to JACKS for four alternative analysis methods (x-axis) on two different libraries (panels) - those that were not used to determine the essential gene set. Every marker represents one cell line or time point sample; median increase is marked with a dark blue line, and estimated distributions are shaded in.

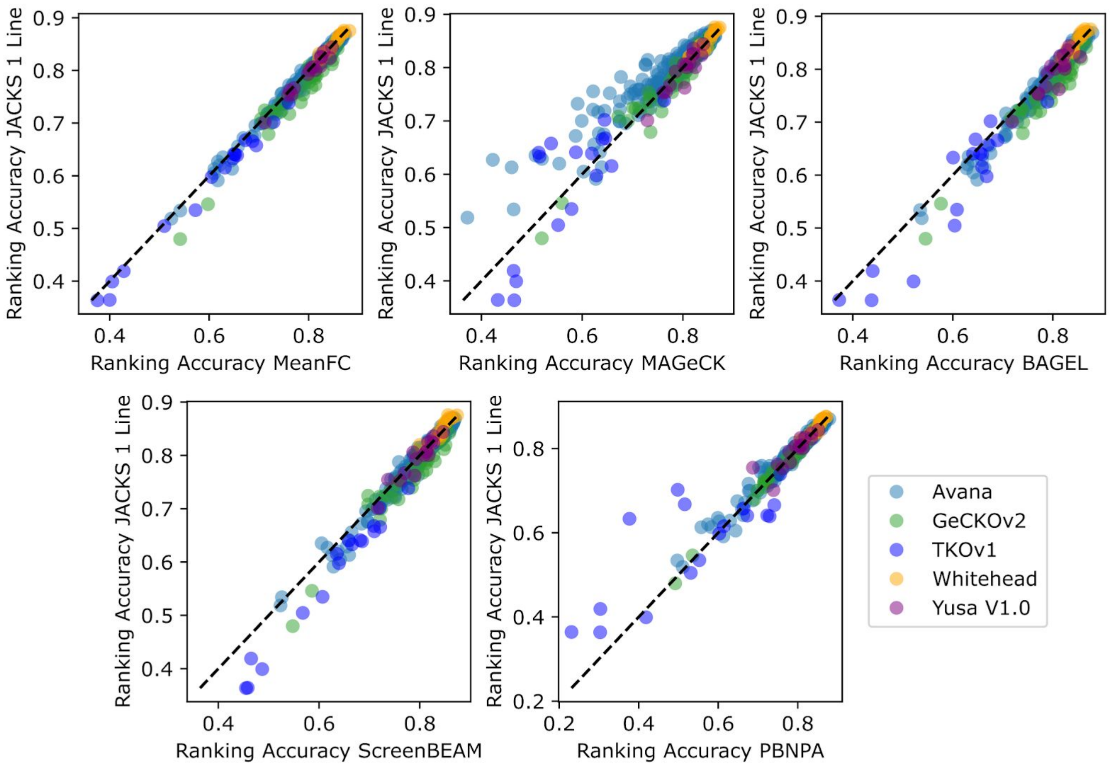

Figure S11: JACKS on only one line at a time performs comparably with existing single-line alternatives when measured by partial 0.2 AUC. Axes, columns, colors as in Figure S6.

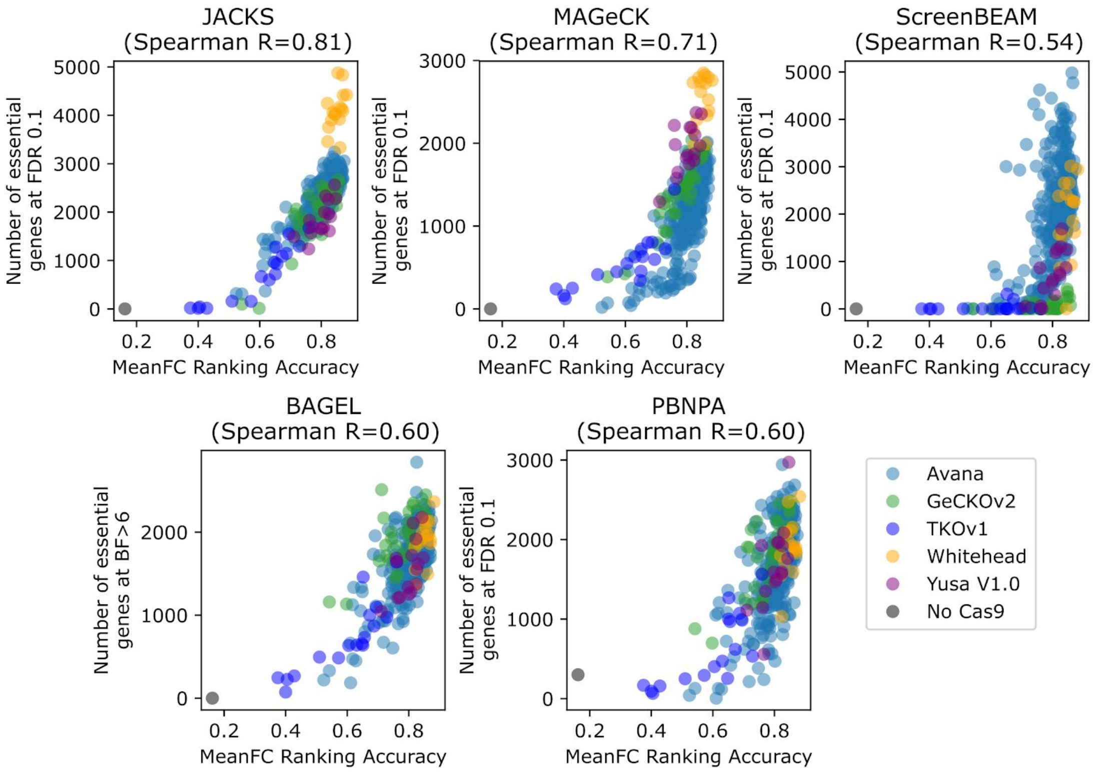

Figure S12: Quantity of significant hits identified by JACKS correlates better with screen quality, as measured by signal in raw gRNA counts. Number of essential genes identified (y-axis) at a false discovery rate of 0.1 (or with a Bayes Factor greater than 6 for BAGEL), compared to the 0.2 pAUC of the MeanFC method (mean fold change of guides targeting each gene) in ranking genes for each screen (x axis), for 5 datasets (colours) each containing many screens (markers), and one screen in HT29 without Cas9 (grey).

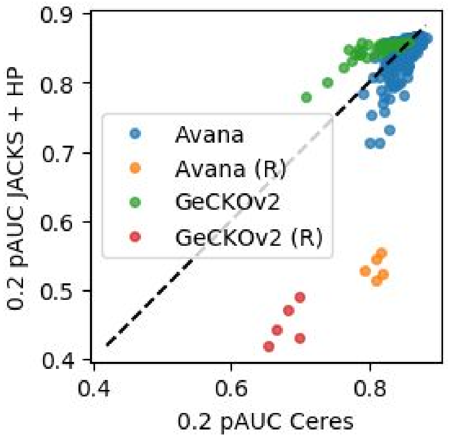

Figure S13: CERES and JACKS(HP) both identify essential genes from random data. Ranking accuracy of CERES (x-axis) compared to JACKS(HP) (y-axis) on Avana (blue) and GeCKOv2 (green) libraries. Each marker corresponds to one cell line, with five randomized experiments (yellow and red markers) included for comparison. Dashed line, y=x.

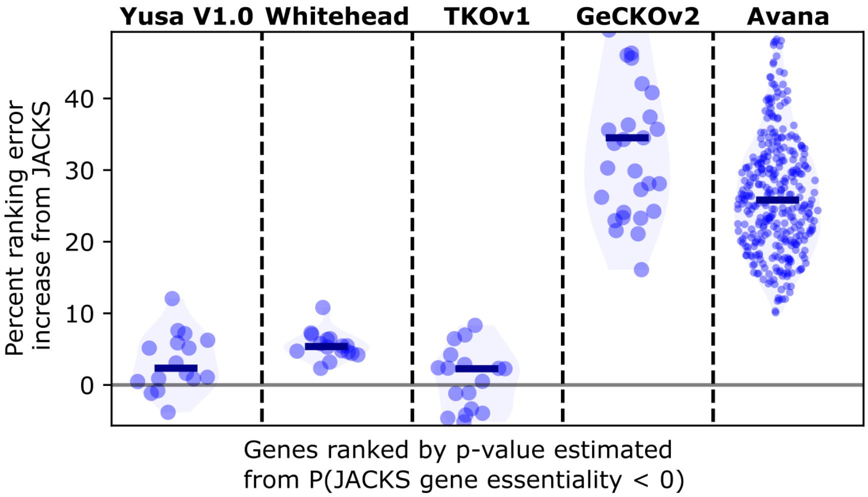

Figure S14: Ranking genes by their posterior probability of a non-zero JACKS gene essentiality effect gives poorer results than ranking by effect size. Percent ranking error increase (y-axis) compared to JACKS for when ranking by variance-derived p-value (x-axis) on five different libraries (panels). Every marker represents one cell line or time point sample; median increase is marked with a dark blue line, and estimated distributions are shaded in. Note that for GeCKO and Avana, JACKS variance estimates shrink as the increased numbers of cell lines make it more sure of relative guide efficacies. This causes the posteriors of essentiality estimates to become confident accordingly, and many p-values shrink. Therefore, rankings computed by this measure focus more on significance and less on effect size, giving poorer results. The alternative p-value determination we use preserves the effect-size ranking, and so does not have this problem.

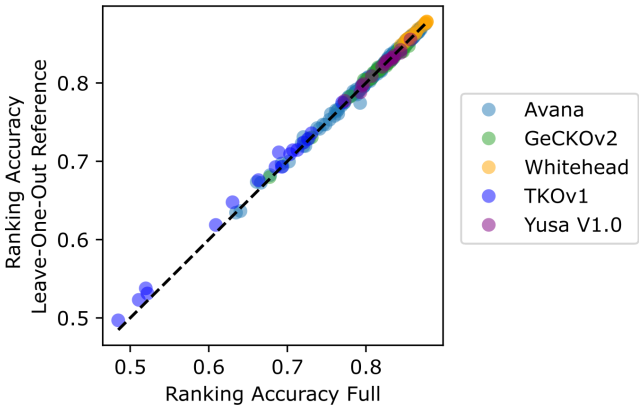

Figure S15: JACKS estimates of gene essentiality can be obtained rapidly using precomputed gRNA efficacies. Recovery accuracy (0.2 pAUC) for full JACKS results (computed on all available data) for a single cell line (x-axis) are nearly identical to those obtained by computing essentialities for a single line at a time, using gRNA efficacies estimated from the rest of the lines (y-axis) for the three considered libraries (colours).
